# Supplementary figures and images for: A novel pathogenesis concept of biliary atresia approached by combined molecular strategies
Source: PLoS One. 2022 Nov 9;17(11):e0277334. doi: 10.1371/journal.pone.0277334 (PMC9645613; doi:10.1371/journal.pone.0277334)

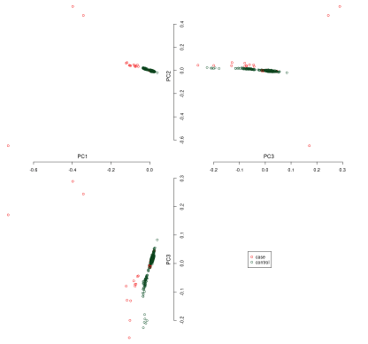

Supplement: S1 Fig — (TIF) [file pone.0277334.s001.tif]
