# Supplementary material for: A novel pathogenesis concept of biliary atresia approached by combined molecular strategies
Source: PLoS One. 2022 Nov 9;17(11):e0277334. doi: 10.1371/journal.pone.0277334 (PMC9645613; doi:10.1371/journal.pone.0277334)
Supplement: S1 Table — (DOCX) [file pone.0277334.s002.docx]

**S1 Table.** Types of progressive familial intrahepatic cholestasis and reported candidate genes.

| **PFIC Type** | **Associated gene (chromosome)** | **Reference** |
| --- | --- | --- |
| PFIC type 1 | ATP8B1 (18q21) | Klomp et al,etc 2004 |
| PFIC type 2 | ABCB11 (2q31) | Pawlikowska et al, 2010 |
| PFIC type 3 | ABCB4 (7q21) | de Vree et al, 1998 |
| PFIC type 4 | TJP2 (9q21) | Sambrotta et al, 2014 |
| PFIC type 5 | NR1H4 (12q23) | Gomez-Ospina et al, 2016 |
| PFIC type 6 | MYO5B (18q21) | Gonzales et al, 2017 |
