# Supplementary material for: A novel pathogenesis concept of biliary atresia approached by combined molecular strategies
Source: PLoS One. 2022 Nov 9;17(11):e0277334. doi: 10.1371/journal.pone.0277334 (PMC9645613; doi:10.1371/journal.pone.0277334)
Supplement: S2 Table — (DOCX) [file pone.0277334.s003.docx]

**S2 Table.** Syndromes in which BA is one of the phenotypes.

| Syndrome | Associated gene (chromosome) | Reference |
| --- | --- | --- |
| Mitchell–Riley syndrome (MTCHR) | RFX6 (6q22.1) | Mitchell et al., 2004 |
| Fanconi anemia, complementation group Q (FANCQ) | ERCC4 (16p13) | Bogliolo et al, 2013 |
| Zimmermann–Laband syndrome 1 (ZLS1) | KCNH1 (1q32) | Balasubramanian and Parker, 2010 |
| Kabuki syndrome 1 (KABUK1) | MLL2 (12q13) | McGaughran et al., 2000 and van Haelst et al., 2000 |
| Alagille syndrome 1 (AGS1) | JAG1 (20p12) | Raymond et al, 1989 |
| Alagille syndrome 2 (AGS2) | NOTCH2 (1p12) | McDaniel et al., 2006 |
