# Supplementary material for: A novel pathogenesis concept of biliary atresia approached by combined molecular strategies
Source: PLoS One. 2022 Nov 9;17(11):e0277334. doi: 10.1371/journal.pone.0277334 (PMC9645613; doi:10.1371/journal.pone.0277334)
Supplement: S3 Table — (DOCX) [file pone.0277334.s004.docx]

**S3 Table.** Syndromes with hyperbilirubinemia**.**

| **Syndrome** | **Associated gene (chromosome)** | **Reference** |
| --- | --- | --- |
| Crigler–Najjar syndrome type I | UGT1A1 (2q37) | Moghrabi et al, 1993 |
| Crigler–Najjar syndrome type II | UGT1A1 (2q37) | Moghrabi et al, 1993 |
| Gilbert (Arias) syndrome | UGT1A1 (2q37) | Koiwai et al., 1995 |
| Dubin–Johnson syndrome | ABCC2 (10q24) | Kajihara et al, 1998 |
| Rotor syndrome | SLCO1B1 (12p12)  SLCO1B3 (12p12) | van de Steeg et al, 2012 |
