# Supplementary material for: A novel pathogenesis concept of biliary atresia approached by combined molecular strategies
Source: PLoS One. 2022 Nov 9;17(11):e0277334. doi: 10.1371/journal.pone.0277334 (PMC9645613; doi:10.1371/journal.pone.0277334)
Supplement: S4 Table — (DOCX) [file pone.0277334.s005.docx]

**S4 Table.** Significant enrichment pathways from the GO biological process database**.**

| **Term** | **Overlap** | **Adjusted P-value** | **Odds Ratio** | **Genes** |
| --- | --- | --- | --- | --- |
| skin development (GO:0043588) | 6/80 | 0.007 | 14.874 | *RYR1, FLG, JAG1, COL1A2, KRT10, ALOXE3* |
| cilium assembly (GO:0060271) | 7/314 | 0.007 | 6.194 | *RP1, TTC8, FAM161A, DNAH5, ARMC9, PCNT, SDCCAG8* |
| cilium organization (GO:0044782) | 5/228 | 0.014 | 6.746 | *TTC8, FAM161A, DNAH5, ARMC9, PCNT* |
| actin-myosin filament sliding (GO:0033275) | 3/38 | 0.014 | 21.232 | *TNNT1, NEB, TTN* |
| muscle filament sliding (GO:0030049) | 3/38 | 0.014 | 21.232 | *TNNT1, NEB, TTN* |
| phototransduction, visible light (GO:0007603) | 2/15 | 0.015 | 44.761 | *RP1, PDE6A* |
| muscle contraction (GO:0006936) | 5/129 | 0.016 | 8.926 | *RYR1, MYOM1, TNNT1, NEB, TTN* |
| plasma membrane-bounded cell projection assembly (GO:0120031) | 5/278 | 0.024 | 5.483 | *TTC8, FAM161A, DNAH5, ARMC9, PCNT* |
| sensory perception (GO:0007600) | 4/51 | 0.024 | 15.349 | *TTC8, ADGRV1, USH1C, ALOXE3* |
| sensory perception of light stimulus (GO:0050953) | 4/95 | 0.024 | 10.090 | *CRYGD, ADGRV1, ROM1, USH1C* |
| inner ear morphogenesis (GO:0042472) | 3/24 | 0.035 | 25.566 | *CHD7, USH1C, SOX9* |
| retinal cone cell development (GO:0046549) | 2/6 | 0.039 | 88.759 | *RP1, USH1C* |
| retinal cone cell differentiation (GO:0042670) | 2/6 | 0.039 | 88.759 | *RP1, USH1C* |
| complement activation, alternative pathway (GO:0006957) | 2/7 | 0.044 | 71.004 | *CR2, CFHR5* |
| parallel actin filament bundle assembly (GO:0030046) | 2/7 | 0.044 | 71.004 | *USH1C, ESPN* |
| positive regulation of cardiac epithelial to mesenchymal transition (GO:0062043) | 2/7 | 0.044 | 71.004 | *JAG1, TGFBR2* |
| peptide cross-linking (GO:0018149) | 3/31 | 0.044 | 19.168 | *FLG, KRT10, TGM4* |
| organelle assembly (GO:0070925) | 6/425 | 0.044 | 4.012 | *ANLN, TTC8, FAM161A, DNAH5, ARMC9, PCNT* |
