# Supplementary material for: A novel pathogenesis concept of biliary atresia approached by combined molecular strategies
Source: PLoS One. 2022 Nov 9;17(11):e0277334. doi: 10.1371/journal.pone.0277334 (PMC9645613; doi:10.1371/journal.pone.0277334)
Supplement: S5 Table — (DOCX) [file pone.0277334.s006.docx]

**S5 Table**. Significant enrichment pathways from Human WikiPathway database**.**

| **Term** | **Overlap** | **Adjusted P-value** | **Odds Ratio** | **Genes** |
| --- | --- | --- | --- | --- |
| Striated Muscle Contraction Pathway (WP383) | 4/38 | <0.000 | 27.597 | *MYOM1, TNNT1, NEB, TTN* |
| Joubert Syndrome (WP4656) | 4/76 | <0.000 | 15.727 | *TTC8, ARMC9, PDE6A, PCNT* |
| Ciliopathies (WP4803) | 5/183 | 0.001 | 8.501 | *RP1, TTC8, FAM161A, KIAA0753, SDCCAG8* |
| Arrhythmogenic Right Ventricular Cardiomyopathy (WP2118) | 4/74 | 0.003 | 13.174 | *LAMA2, SGCB, CACNA2D2, DSC2* |
