# Supplementary material for: A novel pathogenesis concept of biliary atresia approached by combined molecular strategies
Source: PLoS One. 2022 Nov 9;17(11):e0277334. doi: 10.1371/journal.pone.0277334 (PMC9645613; doi:10.1371/journal.pone.0277334)
Supplement: S6 Table — (DOCX) [file pone.0277334.s007.docx]

**S6 Table.** Significant enrichment pathways from Human Reactome database**.**

| **Term** | **Overlap** | **Adjusted  P-value** | **Odds Ratio** | **Genes** |
| --- | --- | --- | --- | --- |
| Striated Muscle Contraction Homo sapiens (R-HSA-390522) | 3/34 | 0.015 | 24.068 | *TNNT1, NEB, TTN* |
| Muscle contraction Homo sapiens (R-HSA-397014) | 6/196 | 0.024 | 6.818 | *RYR1, TNNT1, CACNA2D2, NEB, KCNJ2, TTN* |
