# Supplementary material for: A novel pathogenesis concept of biliary atresia approached by combined molecular strategies
Source: PLoS One. 2022 Nov 9;17(11):e0277334. doi: 10.1371/journal.pone.0277334 (PMC9645613; doi:10.1371/journal.pone.0277334)
Supplement: S7 Table — (DOCX) [file pone.0277334.s008.docx]

**S7 Table.** Results of burden analysis computed by rvtests software.

| **Gene** | **Range** | **Total cases** | **N00** | **N01** | **N10** | **N11** | **P-value** | **FDR** |
| --- | --- | --- | --- | --- | --- | --- | --- | --- |
| ***CCDC8*** | chr19:46410328-46413564 | 414 | 342 | 66 | 0 | 6 | 0.000 | 0.005 |
| ***SYNE2*** | chr14:63853003- 64226449 | 414 | 341 | 72 | 1 | 0 | 1.000 | 1.000 |
| ***BCKDHA*** | chr9:41397817- 41425002 | 414 | 335 | 70 | 7 | 2 | 0.636 | 1.000 |
| ***TGM6*** | chr20:2380900- 2432753 | 414 | 337 | 72 | 5 | 0 | 1.000 | 1.000 |
| ***CHD8*** | chr14:21385198- 21456123 | 414 | 341 | 72 | 1 | 0 | 1.000 | 1.000 |

**Abbreviation**: FDR; false discovery rate
