# Supplementary material for: A novel pathogenesis concept of biliary atresia approached by combined molecular strategies
Source: PLoS One. 2022 Nov 9;17(11):e0277334. doi: 10.1371/journal.pone.0277334 (PMC9645613; doi:10.1371/journal.pone.0277334)
Supplement: Attachment — Submitted filename: Rebutal_PONE-D-22-12933_091065.docx [file pone.0277334.s009.docx]

**PLOS ONE**

**Dear Editor-in-chief,**

The authors thank all reviewers for their valuable suggestion for the manuscript PONE-D-22-12933 entitled “A novel pathogenesis concept of biliary atresia approached by combined molecular strategies”. The authors have carefully read the suggestion and improved the manuscript accordingly.

**Our response to the comments of the reviewer:**

All authors have read and approved of these revisions, which we hope they would meet with your approval following answer statements in below:

**Reviewer #2**

*1. As shown in Table 2, of the 75 enrolled cases, twenty-two cases with genetic variants which were related to cholestatic disorders were identified. It may be an important finding to identify the variants in the related cholestatic jaundice genes in biliary atresia frequently. On the other hand, it is important to show the diagnosis of biliary atresia is correctly made in the subject cases. The authors should show that the genetic abnormality overlaps in the biliary atresia cases after other cholestatic diseases were correctly differentiated.*

**Answer:** The diagnosis of biliary atresia cases in this study was deepened on intraoperative cholangiography and intraoperative findings together with additional conformation by pathological report and clinical follow up. (Page5 Line7)

*2. Based on this point of view, the authors should present the anatomical pattern of biliary atresia studied in these cases, referring to the following article. (1: Anatomical patterns of biliary atresia including hepatic radicles at the porta hepatis influence short- and long-term prognoses. Pancreat Sci. 2021 Nov;28(11):931-941., 2: The anatomic pattern of biliary atresia identified at the time of Kasai hepatoportoenterostomy, and early postoperative clearance of Jaundice are significant predictors of transplant-free survival. Ann Surg. 2011 Oct; 254(4): 577-585.)*

**Answer:** We already added anatomical type of biliary atresia identified intraoperatively in our manuscript. The majority of anatomy was type III which had fibrosis at porta hepatis (68 cases, 90.67%). After we included the anatomical factors into analysis of multivariate cox proportional hazards model, the results revealed independent correlation increasing hazard risk in BA cases whose age at surgery was more than 90 days and those with identified variant status. In addition, we also cited two suggestion articles in the materials and method section (Page6 Line10). With this addition, the result was reported in Table1.

*3. In addition, the authors can demonstrate that you have correctly diagnosed cholestatic diseases when the authors show the signs of Alagille syndrome and the findings of liver biopsy.*

**Answer:** The exclusion of other syndromes which clinical features similar to biliary atresia e.g., Alagille syndrome were performed by histopathology that revealed biliary hypoplasia, paucity of bile duct. (Page5 Line9)

*4. In addition, the authors conducted a multivariate analysis, including the day of surgery and variant status. Since it has been shown that the anatomical pattern of biliary atresia also has a significant impact on prognosis, we believe that a multivariate analysis, including these factors, would provide important insights.*

**Answer:** Together with the second question, we also included the anatomical factors into cox proportional hazards model calculation. The factors of age at Kasia operation and identified variant status were remained independently significantly increasing hazard risk. (Fig 5)

Attached with this mail, we submit the first revision of our manuscript. We are looking forward to hearing from the PLOS ONE.

Sincerely yours,

Surasak Sangkhathat, M.D., Ph.D.

Department of Surgery,

Faculty of Medicine, Prince of Songkla University,

Thailand 90110
